# The Assembly of MXenes from 2D to 3D

**DOI:** 10.1002/advs.201903077

**Published:** 2020-02-13

**Authors:** Zhitan Wu, Tongxin Shang, Yaqian Deng, Ying Tao, Quan‐Hong Yang

**Affiliations:** ^1^ Nanoyang Group State Key Laboratory of Chemical Engineering School of Chemical Engineering and Technology Tianjin University Tianjin 300350 China; ^2^ Joint School of National University of Singapore and Tianjin University International Campus of Tianjin University Binhai New City Fuzhou 350207 China; ^3^ Shenzhen Key Laboratory for Graphene‐based Materials Engineering Laboratory for Functionalized Carbon Materials Graduate School at Shenzhen Tsinghua University Shenzhen 518055 China

**Keywords:** applications, assembly, macroscopic structures, MXenes

## Abstract

Since their discovery in 2011, transition metal carbides or nitrides (MXenes) have attracted a wide range of attention due to their unique properties and promise for use in a variety of applications. However, the low accessible surface area and poor processability of MXene nanosheets caused by their restacking have severely hindered their practical use, and this is expected to be solved by integrating them into macroscopic assemblies. Here, recent progress in the construction of MXene assemblies from 2D to 3D at the macro and/or microlevel is summarized. The mechanisms of their assembly are also discussed to better understand the relationship between performance and assembled structure. The possible uses of MXene assemblies in energy conversion and storage, electromagnetic interference shielding and absorption, and other applications are summarized.

## Graphical abstract

The MXene assemblies are promising for broadening the applications of MXenes and promoting their realization in practice. Herein, the MXene assemblies from 2D to 3D at macro and/or microlevels are reviewed. The corresponding assembly mechanisms and applications are also summarized to better deepen the cognition of relationships between assembled structures and specific applications.

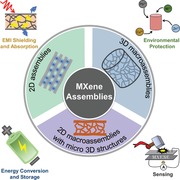

## 1 Introduction

MXene, a new member of the 2D materials family, is a kind of metal carbide or nitride material with a 2D layer structure.[qv: 1] In 2011, Gogotsi's group first succeeded in etching the Al layers from Ti_3_AlC_2_ with hydrofluoric acid (HF), resulting in Ti_3_C_2_ MXene.[qv: 2] Subsequently, over 30 kinds of MXenes, including Ti_2_C, Ta_4_C_3_, (Ti_0.5_,Nb_0.5_)_2_C, (V_0.5_,Cr_0.5_)_3_C_2_, Ti_3_CN,[qv: 3] Nb_2_C, V_2_C, and Mo_2_C,[qv: 4,5] have been produced by the selective etching of A elements from the corresponding MAX phases with different etchants, such as HF,[qv: 2] ammonium hydrogen difluoride (NH_4_HF_2_),[qv: 6] a mixture of acid and fluoride salt,[qv: 7] etc. All these MXenes can be denoted with the formula M*_n_*_+1_X*_n_*T*_x_* (*n* 1–3), where M is an early transition metal (Sc, Ti, V, Cr, Zr, Nb, Mo, Hf, Ta, or W), X is carbon and/or nitrogen, and T*_x_* stands for a surface functional group (—O, —OH, and/or —F, etc.).[qv: 8]

MXenes have a lattice with hexagonal lattice symmetry inherited from their parent MAX phase and have a superior electrical conductivity (6000–8000 S cm^−1^) due to their metallic backbone.[qv: 1,8] Abundant surface functional groups provide a large number of active sites on the surface of MXenes, and this has tremendous potential for surface modification and the highly efficient loading of active substances.[qv: 9–12] Additionally, MXenes have good thermal conductivity,[qv: 6] a tunable bandgap and excellent mechanical strength,[qv: 13,14] thus having great prospects in the fields of energy conversion and storage,[qv: 15–17] electromagnetic interference (EMI) shielding and absorption,[qv: 18–20] sensing,[qv: 21,22] environmental protection, and so on.[qv: 23,24]

However, similar to other 2D materials, MXenes also have a propensity for “face to face” stacking and aggregation due to strong van der Waals forces, which greatly limits their performance in practical applications.[qv: 25,26] Recently, to address the stacking problem for a high surface utilization and obtain functional MXene materials with well‐tailored structures, intense efforts from different aspects, such as surface modification,[qv: 27] heteroatom doping,[qv: 28] buckling and crumpling,[qv: 29–32] introducing interlayer spacers,[qv: 33] templating,[qv: 34] and crosslinking[qv: 35] have been made.

Ways of assembling MXenes into 3D structures at the macro and/or microlevel are greatly needed to overcome the restacking problem of 2D materials, and have been extensively studied.[qv: 18,36,37] The 2D MXene nanosheets can be used as building blocks to construct MXene assemblies with desired structures by well‐designed methods. Their excellent properties are expected to be inherited by the assemblies, thus essentially expanding their applications. Hence, there is a great need to summarize recent progress in the design of MXene assemblies for diverse applications.

**Figure 1:**
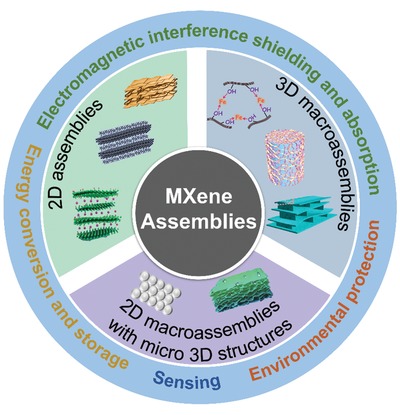
MXene assemblies from 2D to 3D at the macro and/or microlevel. Reproduced with permission.[qv: 18,33,34,37,42–45] Reproduced with permission.[qv: 46,47] Copyright 2016 and 2018, Royal Society of Chemistry.

Previously, several reviews on the synthesis, properties, and applications of MXenes have been published.[qv: 8,15,25,38–41] However, none of them have summarized the latest advances on MXene from an assembly perspective. A timely and focused progress report of MXene assemblies is expected to further accelerate the development of these emerging materials and promote their applications. Here, recent efforts on MXene assemblies and the corresponding assembly strategies are reviewed. To facilitate the discussion, the assemblies are classified into three categories according to their dimensional structures at the macro and/or microlevels. These are, 2D assemblies, 2D macroassemblies with micro 3D structures, and 3D macroassemblies (**Figure** **1**). We then emphasize the latest progress on MXene assemblies for different applications, including energy conversion and storage, EMI shielding, and absorption, etc. Finally, the challenges and prospects in the development of MXene assemblies are highlighted.

## 2 The MXene Assemblies

In this section, we present the recent progress on the production of MXene assemblies from 2D to 3D at the macro and/or microlevel. They can be grouped into three categories: 2D assemblies, 2D macroassemblies with micro 3D structures, and 3D macroassemblies. The assembly mechanisms are also discussed in order to understand the assembly processes.

### 2.1 2D Assemblies

**Figure 2:**
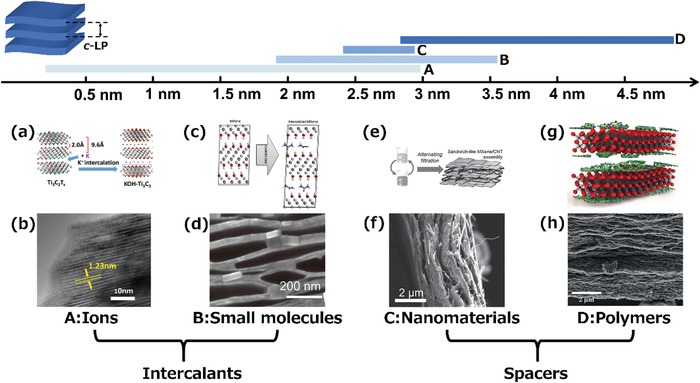
The *c*‐lattice parameter ranges of 2D MXene assemblies with different external components. a) Schematic of ion‐intercalated MXenes. b) A TEM image of a K ion‐intercalated MXene.[qv: 57] c) Schematic of a small molecule intercalated MXene. d) SEM image a MXene cointercalated with hydrazine monohydrate (HM) and N,N‐dimethylformamide (DMF). Reproduced with permission.[qv: 54] Copyright 2013, Springer Nature. e) Schematic of an alternating filtration process to fabricate Ti_3_C_2_T*_x_*/CNT composites. f) SEM image of Ti_3_C_2_T*_x_*/CNT composite.[qv: 43] g) Schematic of Ti_3_C_2_T*_x_*‐PANI hybrid films. h) Cross‐sectional SEM image of Ti_3_C_2_T*_x_*‐PANI hybrid films. Reproduced with permission.[qv: 58] Copyright 2018, Royal Society of Chemistry.

The coupling phenomenon of 2D nanosheets is common for 2D materials.[qv: 48–50] In particular, MXene nanosheets are easy to aggregate or restack due to strong van der Waals forces between them, leading to the loss of accessible surface area and active sites.[qv: 29] To address this issue, introducing an external component between the nanosheets is a general strategy,[qv: 51–53] and those commonly used are ions,[qv: 36] small molecules,[qv: 54] nanomaterials, and polymers.[qv: 55,56] Various 2D MXene assemblies can be achieved by introducing different types of external components with different sizes and interactions between the layers so that the *c*‐lattice parameter (*c*‐LP) of 2D MXene assemblies ranges from ≈0.3 to 4.8 nm (**Figure** **2**).

Depending on their interaction with the MXene sheets and the assembly mechanism, the external components are mainly divided into two types, intercalants and spacers. Among them, ions and small molecules are classified as intercalants, while nanomaterials and polymers are classified as spacers. The role of intercalants is mainly to intercalate the accordion‐like multilayer MXenes (m‐MXenes), thereby expanding the interlayer spacing or delaminating them into monolayers or a few layers. The function of spacers is mainly to prevent the MXene nanosheets from restacking during the assembly of delaminated MXenes (d‐MXenes).

#### 2.1.1 Ions and Small Molecules

**Figure 3:**
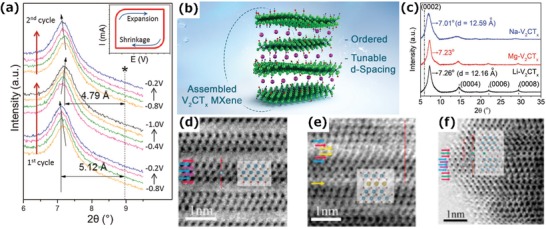
a) Electrochemical in situ XRD study of multilayer exfoliated Ti_3_C_2_T*_x_* in a 1 m KOH solution. Reproduced with permission.[qv: 36] Copyright 2013, The American Association for the Advancement of Science. b) Schematic of the cation‐driven assembly process used for the fabrication of ordered and highly stable V_2_CT*_x_* flakes. c) XRD patterns of films fabricated using flakes assembled with different cations.[qv: 33] d–f) Annular bright‐field (ABF) images of Ti_3_C_2_T*_x_*, Na*_x_*Ti_3_C_2_T*_x_*, and Al*_x_*Ti_3_C_2_T*_x_*. Reproduced with permission.[qv: 64] Copyright 2015, American Chemical Society.

The intercalation of ions or small molecules between MXene layers is a common strategy for regulating the structure of 2D assemblies, which generally results in a more stable 2D structure with a larger or smaller *c*‐LP than the pure MXene.[qv: 59] The spontaneous intercalation of cations (such as Li^+^, Na^+^, K^+^, NH_4_^+^, Mg^2+^, Al^3+^) from aqueous solutions between 2D MXene layers was first demonstrated by Lukatskaya et al.[qv: 36] As shown in **Figure** **3**a, when a Ti_3_C_2_T*_x_* electrode was cycled in a KOH‐containing electrolyte, there was a downshift of the (0002) peak position in the X‐ray diffraction (XRD) pattern, which indicates an increase of interlayer spacing. The intercalation behavior was also influenced by the pH value. For example, a larger interlayer spacing was achieved in the case of high‐pH solutions, such as KOH, NH_4_OH, NaOH, and LiOH. The higher pH value leads to a higher —OH content by replacing —F with —OH,[qv: 11] which may be beneficial for cation adsorption and diffusion between the MXene layers.[qv: 60,61] Meanwhile, the surface of the MXene is negatively charged due to its surface functional groups, resulting in significant electrostatic interaction between the cations and MXenes, which accounts for the improved stability of ion‐intercalated MXenes.[qv: 62]

Ions can also induce the assembly of d‐MXene, resulting in an ordered and stable 2D structure. Highly stable and flexible multilayer films of V_2_CT*_x_* and Ti_2_CT*_x_* MXenes derived from their corresponding chemically unstable d‐MXene flakes have been formed by a cation‐driven (Li^+^, Na^+^, Mg^2+^) assembly process.[qv: 33] The as‐prepared films have a lower *c*‐LP than the pristine films (Figure 3b,c), which is due to the strong electrostatic interaction between the cations and the flakes. In addition, the transition metal species are in a more reduced state because of charge transfer from the inserted alkali cations to the flakes, which leads to high chemical stability and excellent electrochemical performance. Similarly, Chen et al. reported a pristine Ti_3_C_2_T*_x_* film with significantly enhanced conductivity, mechanical strength, and environmental stability due to the removal of extrinsic intercalants, which is achieved by the proton acid colloidal processing approach.[qv: 63]

To further understand the intercalation mechanism of MXenes, numerous efforts have been devoted to exploring the intercalation of Ti_3_C_2_T*_x_*. Wang et al. pointed out that Na^+^ and Al^3+^ can be electrochemically intercalated or deintercalated between the layers of Ti_3_C_2_T*_x_* flakes with different intercalation behaviors.[qv: 64] As shown in Figure 3d–f, Na^+^ forms double Na‐atomic layers, and the intercalated Na^+^ prefers to occupy the sites on top of C‐atoms, while, the Al^3+^ forms a single Al‐atomic layer, occupying the sites on top of C‐atoms in one Ti_3_C_2_T*_x_* layer and close to the sites on top of Ti‐atoms in the other layer, leading to the horizontal sliding of Ti_3_C_2_T*_x_* monolayers. Eames and Islam found that a greater amount of Li^+^ or Mg^2+^ than Na^+^ or K^+^can be intercalated into MXenes, which is related to the size of intercalated ions.[qv: 65]

Ion intercalation can also be achieved by ion‐exchange, which also enables regulation of the MXene interlayer spacing.[qv: 24,66] Ion‐exchange was demonstrated in Li^+^ intercalated MXene by Ghidiu et al.[qv: 66] Similar to swelling clay minerals and metal dichalcogenides, it was found that the interlayer spacing of the ion‐exchanged material increases stepwise with increased humidity because of water intercalation. As mentioned above, Naguib and Gogotsi have reported that —F and —OH groups on the surface of alkali‐treated Ti_3_C_2_ (alk‐Ti_3_C_2_) cause the surface to be negatively charged and as a result ion‐exchange occurs when alk‐Ti_3_C_2_ is immersed in a solution containing metal cations because some metal cations are adsorbed by electrostatic interaction.[qv: 62] Similarly, Peng et al. have shown that after cation intercalation, alk‐Ti_3_C_2_T*_x_* adsorbs Pb^2+^, which is due to the strong metal–ligand interaction between Pb^2+^ and [Ti‐O]‐H^+^ as well as the ion‐exchange behavior of Pb^2+^ and [Ti‐O]‐H^+^/[Ti‐O]‐Na^+^ in 2D layered alk‐MXene with a large surface area.[qv: 24] Similarly, Co^2+^ intercalated V_2_C MXene has been fabricated by ion‐exchange because of the strong electrostatic interaction between the Co^2+^ and the MXene flakes, resulting in a controllable interlayer spacing and a more stable structure due to the formation of strong V—O—Co bonding.[qv: 67] At the same time, this method is also suitable for other ions. For example, the intercalation of Sn^4+^ and Se^4+^ has also been recently achieved by ion‐exchange.[qv: 68,69]

**Figure 4:**
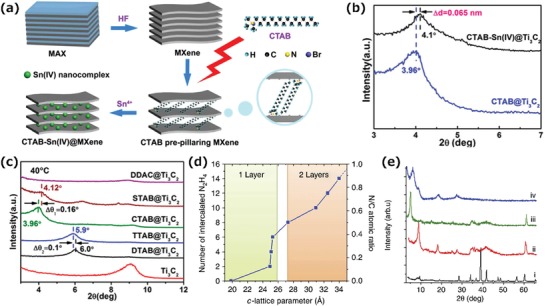
a) Schematic of the preparation of CTAB‐Sn (IV)@Ti_3_C_2_. b) XRD patterns of CTAB@Ti_3_C_2_ before and after Sn^4+^ intercalation. c) XRD patterns of cationic surfactants prepillaring Ti_3_C_2_ at 40 °C. Reproduced with permission.[qv: 70] Copyright 2017, American Chemical Society. d) Change in m‐MXene *c*‐LP as a function of the number of N_2_H_4_ intercalated molecules. e) XRD patterns of (i) pristine, (ii) exfoliated, (iii) DMSO‐intercalated and (iv) delaminated Ti_3_C_2_T*_x_*. Note the disappearance of the non‐basal peaks at ≈60° in (iv). Reproduced with permission.[qv: 54] Copyright 2013, Springer Nature.

A stable structure of pillared MXene with a tunable interlayer spacing was produced by a pillaring method using cationic surfactants as prepillaring agents. The reaction is driven by electrostatic interactions and has promise for rapid intercalation and deintercalation of ions. For instance, a pillared Ti_3_C_2_T*_x_* MXene was produced by the spontaneous intercalation of cetyltrimethylammonium bromide (CTAB) in the liquid‐phase with the assistance of electrostatic interactions between MXene and surfactant cations.[qv: 70] The intercalation of Sn^4+^ achieved by ion‐exchange results in pillared CTAB‐Sn (IV)@Ti_3_C_2_, which has a smaller interlayer spacing, as confirmed by XRD (**Figure** **4**a,b). As a result, the CTAB‐Sn (IV)@Ti_3_C_2_ showed good cycling and rate performance because the pillared nanostructure has a large spacing for Sn^4+^ intercalation and thereby resists the diffusion‐controlled process during lithiation/delithiation.[qv: 71] Moreover, the interlayer spacing can be tuned in the range from 1 to 2.708 nm by changing the type of surfactant used and the alkyl chain length, as well as the temperature (Figure 4c). The tuning of the interlayer spacing plays an important role in matching the interlayer spacing of MXenes with the sizes of different ions so as to maximize the performance of the MXenes. Xu et al. have reported the preintercalation of CTAB into Ti_3_C_2_T*_x_* to realize the reversible insertion of Mg^2+^, thus achieving excellent volumetric performance and rate capability for magnesium ion batteries, and this indicates the possible wide use of surfactant preintercalation in the field of energy conversion and storage.[qv: 72] In addition to the surfactants, small molecules like tris(2‐aminoethyl) amine (TAEA) was also reported as the interlayer pillaring component for the layer‐by‐layer self‐assembly of Ti_3_C_2_T*_x_* MXene.[qv: 73]

MXenes can also be intercalated by various small molecules, which can further expand the interlayer spacing of MXene. In a typical example, Mashtalir et al. have demonstrated the spontaneous intercalation of urea, dimethyl sulfoxide (DMSO), and hydrazine, and its cointercalation with N, N‐dimethylformamide, resulting in an increase of the interlayer spacing of Ti_3_C_2_T*_x_* (Figure 4d).[qv: 46,54] In addition, the intercalation of DMSO followed by ultrasonication produces the delamination of multilayer MXene, leading to separated 2D layers (Figure 4e). To reveal the mechanisms of intercalation, Overbury and co‐workers have also explored the intercalation of urea and found that during intercalation, Lewis acid sites, such as H^+^ or Ti (III) in the MXene solutions promote surface‐mediated urea decomposition, hydrolysis of cyanate, and the protonation of NH_3_ to NH_4_^+^.[qv: 74] The active metal sites and strongly acidic/basic moieties serve as catalytic centers to facilitate the decomposition of the intercalants, thereby promoting the intercalation of small molecules.

In addition, the intercalation of small molecules can be facilitated by the preintercalation of ions. For example, the presence of potassium cations between MXene layers improves the intercalation of water, thus increasing the stability of the 2D structure against changes in environmental conditions.[qv: 59] The intercalation of ions can also be facilitated by the preintercalation of small molecules. Lin et al. demonstrated that the intercalation of EMI‐TFSI electrolyte in the presence of water molecules between the MXene layers (MXene hydrogel) leads to an improved capacitance in the EMI‐TFSI electrolyte.[qv: 75] Meanwhile, they found that the elimination of water from the MXene films leads to irreversibly restacking of MXene layers, thus decreasing the interlayer spacing. Therefore, the intercalation of ions and small molecules can be mutually promoted.

#### 2.1.2 Nanomaterials and Polymers

Spontaneous intercalation into m‐MXenes is hard to achieve for nanomaterials and polymers because of their larger size than intercalants. Instead, as spacers to the MXene nanosheets, they can prevent delaminated MXenes from restacking during assembly processes and weaken the interplane attraction, thus increasing the interlayer spacing.

**Figure 5:**
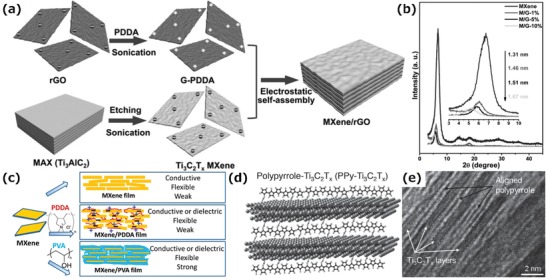
a) Schematic of the synthesis of MXene‐rGO hybrids. b) XRD patterns of the MXene and MXene‐rGO hybrids. Reproduced with permission.[qv: 76] c) Schematic of MXene films, PDDA‐MXene, and PVA‐MXene hybrid films. Reproduced with permission.[qv: 56] Copyright 2014, National Academy of Sciences. d) Schematic of polypyrrole‐MXene hybrid films. e) A cross‐sectional TEM image of aligned polypyrrole chains (bright layers) between MXene sheets (darker layers).[qv: 42]

Nanomaterials, such as carbon nanotubes (CNTs), graphene and MoS_2_, serve as spacers to suppress the aggregation of MXenes,[qv: 43,49,76] thus ensuring their performance for various applications. Among them, achieving a uniform distribution of nanomaterials between the layers is the key point in preventing the MXenes from restacking, which ensures the availability of a larger number of active sites. For instance, a sandwich‐like Ti_3_C_2_T*_x_*/CNT composite paper was fabricated by the alternating filtration of Ti_3_C_2_T*_x_* and CNT dispersions,[qv: 43] which ensured the uniform distribution of CNTs between the MXene layers. As a result, compared with a compact MXene film, the Ti_3_C_2_T*_x_*/CNT composite paper provides easier access to the electrolyte ions while maintaining considerable conductivity, which leads to a better electrochemical performance. Similarly, Cai et al. have reported the production of a stretchable sandwich‐like Ti_3_C_2_T*_x_*/CNT composite film by a layer‐by‐layer spray coating technique, that showed an extraordinary sensing performance.[qv: 55] A 2D/2D integrated structure of MXene/nanomaterial composites has also been proposed recently. RGO/Ti_3_C_2_T*_x_* hybrid films have been fabricated by the vacuum‐assisted filtration of a mixed dispersion of graphene oxide (GO) and Ti_3_C_2_T*_x_*, followed by heat treatment under vacuum for GO reduction, that showed excellent volumetric performance in aqueous acidic electrolytes.[qv: 77] Li et al. have reported the formation of a flexible MXene/graphene film by vacuum filtration of a mixed suspension of MXene and graphene, in which the graphene was synthesized by the electrochemical exfoliation of a graphite foil.[qv: 78] There are some other strategies dedicated to preventing the direct stacking of MXenes, such as the layer‐by‐layer self‐assembly of MXene nanosheets and other nanomaterials. Xie et al. have reported a porous Ti_3_C_2_T*_x_*/CNT composite paper which was assembled using the electrostatic interaction between negatively charged MXene nanosheets and positively charged CNTs modified by CTAB.[qv: 79] The self‐assembly of a MXene/graphene composite has been achieved by Yan et al. by a similar method.[qv: 76] In this study, rGO modified by poly(diallyldimethylammonium chloride) (PDDA) with a positive zeta potential enables the self‐assembly of rGO and MXene layer‐by‐layer by electrostatic interaction, resulting in a well‐aligned structure and increased interlayer spacing, which accounts for its excellent electrochemical performance (**Figure** **5**a,b). Additionally, Zhao et al. have demonstrated the self‐assembly of positively charged N‐rich porous carbon nanosheets modified by PDDA and negatively charged MXene nanosheets with an enlarged interlayer spacing and unique 3D interconnected networks.[qv: 80]

Similarly, polymers can regulate the interlayer spacing of MXenes by forming 2D MXene assemblies, as first reported by Gogotsi's group.[qv: 56] In this work, PDDA‐Ti_3_C_2_T*_x_* and PVA (polyvinyl alcohol)‐Ti_3_C_2_T*_x_* hybrid films were assembled by the electrostatic interaction or hydrogen bonding between Ti_3_C_2_T*_x_* and the polymer, which resulted in the confinement of the polymer between the MXene layers (Figure 5c). The interlayer spacing of MXenes with the polymer inserted is larger than that MXene assemblies with intercalants. Moreover, compositing with a polymer gives the MXene assemblies excellent tensile strength while maintaining considerable conductivity, which has great potential for their use as flexible devices, and for energy conversion and storage, and radiofrequency shielding, etc. In the same way, Shahzad et al. have fabricated a flexible Ti_3_C_2_T*_x_*‐sodium alginate (SA) film by vacuum filtrating a mixed solution of Ti_3_C_2_T*_x_* and SA, which shows excellent an EMI shielding performance.[qv: 19] The formation of the Ti_3_C_2_T*_x_*‐SA film is mainly because of the hydrogen bonding between the functional groups of SA (—OH, —COO, and =O) and the terminal groups of Ti_3_C_2_T*_x_*.

Nevertheless, mixing a MXene colloidal solution with polymers has tremendous drawbacks. Typically, polymers in the solution will aggregate randomly during the vacuum‐assisted filtration process and as a result will cover the surface of the MXenes in a disordered state, thus limiting their performance in various applications.[qv: 81] To address this problem, in situ polymerization on the surface of the MXene was proposed. Boota et al. have demonstrated the in situ polymerization of pyrrole on the surface of Ti_3_C_2_T*_x_* nanosheets in an aqueous solution without an oxidant, thus resulting in a larger interlayer spacing and excellent electrochemical performance (Figure 5d,e).[qv: 42] Specifically, the acidic property of the Ti_3_C_2_T*_x_* surface groups induces the protonation of pyrrole and causes strong interaction between the pyrrole and Ti_3_C_2_T*_x_*, thus facilitating the gradual formation of a dimer, and longer chains. The hydrogen bonding between pyrrole oligomers and the Ti_3_C_2_T*_x_* sheets causes the self‐aligning of polypyrrole (PPy), therefore ensuring a uniform distribution of PPy on the surface. Similarly, MXene hybrid films with poly(3,4‐ethylenedioxythiophene) (PEDOT), polyaniline (PANI) have also been reported, and they are all synthesized using the oxidant‐free and in situ polymerization of monomers on the surface of MXenes, which is more environmentally friendly, safer, and less costly than traditional polymerization methods.[qv: 58,81]

### 2.2 2D Macroassemblies with Micro 3D Structures

Compared with the 2D layer structure, a 3D porous structure is generally more capable of directly overcoming the restacking problem caused by van der Waals forces and hydrogen bonding between the 2D nanosheets.[qv: 82–84] Specifically, 3D assembly enables the crosslinking of MXene nanosheets by introducing a stronger interlayer enlargement effect, which results in a 3D interconnected network of MXenes. Similar to graphene, the formation of 3D networks leads to a higher surface utilization while maintaining the structural merits and unique properties of the 2D MXene nanosheets.[qv: 85]

The 3D assembly of MXene nanosheets can form 2D macroassemblies with micro 3D structures, 3D monoliths, or other macroscopic structures, depending on the selected assembly strategy and the strength of the linkages in the MXene assembly. Among these, 2D macroassemblies with micro 3D structures can be obtained by two strategies: a) 3D assembly to form 2D macroassemblies and b) modification after 2D assembly. As‐prepared 2D macroassemblies with micro 3D structures have a higher utilization of active sites with better functionality than 2D assemblies of MXene, thus playing an important role in constructing functional MXene assemblies.

We shall now summarize the methods for preparing 2D MXene macroassemblies with a 3D structure at microlevel from the two aspects mentioned above, and the corresponding assembly mechanisms will be discussed.

#### 2.2.1 3D Assembly to form 2D Macroassemblies

**Figure 6:**
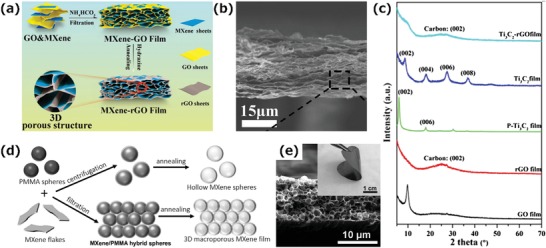
a) Schematic of the fabrication of MXene‐rGO films. b) A cross‐sectional SEM image of the Ti_3_C_2_‐rGO hybrid film. c) XRD patterns of GO, rGO, p‐Ti_3_C_2_ (pure Ti_3_C_2_), Ti_3_C_2_, and Ti_3_C_2_‐rGO films. Reproduced with permission.[qv: 86] Copyright 2018, American Chemical Society. d) Schematic showing the construction of hollow MXene spheres and 3D macroporous MXene frameworks. e) Cross‐sectional SEM image of the 3D macroporous Ti_3_C_2_T*_x_* film. Inset: optical image showing the flexibility of a 3D macroporous Ti_3_C_2_T*_x_* film.[qv: 34]

Inspired by the 3D assembly of GO, Ma et al. developed a new strategy to fabricate porous MXene‐based electrode materials with the addition of GO (**Figure** **6**a).[qv: 86] GO and MXene form a stable homogenous dispersion due to their strong electrostatic repulsion. The addition of ammonium bicarbonate breaks the electrostatic balance, resulting in MXene‐GO hybrid scaffolds. To obtain a MXene‐rGO hybrid film with a porous structure, dehydration by vacuum filtration was used followed by a reduction treatment. During the filtration process, the MXene‐GO hybrid scaffolds were gradually crosslinked, thereby forming the 3D porous composite films (Figure 6b). XRD patterns showed that there is no obvious Ti_3_C_2_T*_x_* peak in MXene‐rGO hybrid films (Figure 6c), which is evidence for the disordered stacking of the MXene nanosheets.

The template method is also a common method for the construction of macroporous structures. A 2D macroscopic MXene film with a hollow spherical framework was produced using sacrificial poly(methyl methacrylate) (PMMA) sphere templates (Figure 6d).[qv: 34] In this work, the PMMA spheres were spontaneously wrapped by MXene flakes as a result of the interaction between their surface hydroxyl groups. A hollow spherical framework was then obtained by removing the PMMA from a 2D macroscopic MXene‐PMMA hybrid film (Figure 6e), whose rough surface gave it good hydrophobicity. At the same time, its microscopic structure was highly tunable, making it have great potential for various applications. Specifically, the wall thickness of the Ti_3_C_2_T*_x_* hollow spheres and the flexibility of the film can be tuned by the mass ratio of PMMA to Ti_3_C_2_T*_x_*, and the size of the spheres can be tuned by the size of PMMA spheres used. The film is also highly conductive due to good contact between the spheres and the metallic nature of the MXenes and has rapid ion transport channels because of the macroporous structure, which accounts for the better electrochemical performance than 2D structural MXene films.

#### 2.2.2 Modification After 2D Assembly

2D MXene macroassemblies with micro 3D structures can also be achieved by modification of 2D assemblies. Specifically, a 2D assembly is first produced followed by treatment to produce micro 3D structures.

**Figure 7:**
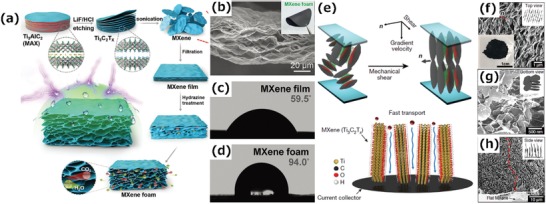
a) Schematic of the fabrication of a hydrophobic and flexible MXene foam. b) Cross‐sectional SEM image of the MXene foam. Inset: optical image showing the flexibility of the foam. c,d) Water contact angle measurements of c) the MXene film and d) the MXene foam.[qv: 18] e) Illustration of the method to fabricate aligned Ti_3_C_2_T*_x_* MXene films. f) Top view of the SEM image of a MXene lamellar liquid crystal (MXLLC). Inset: optical image of the MXLLC. g,h) SEM images of parallel nanosheets perpendicular to the MXene current collector in (e). g) bottom view and h) side view. Reproduced with permission.[qv: 87] Copyright 2018, Springer Nature.

Liu et al. have demonstrated the production of a freestanding, hydrophobic, lightweight, and flexible MXene foam by a hydrazine‐induced foaming process of a MXene film obtained by vacuum filtrating a d‐MXene colloidal solution (**Figure** **7**a).[qv: 18] Compared with compact MXene films, the MXene foams have a continuous cellular structure, resulting in a lower density and a highly porous structure (Figure 7b), which accounts for the excellent EMI shielding performance. Specifically, hydrazine reacts with the oxygen‐containing functional groups of the MXene films and large amounts of gaseous species is released, thereby producing high pressure between the layers to expand the interlayer spacing. The density, thickness, and electrical conductivity of the MXene foams are tunable by changing the amount of hydrazine used. The resulting MXene foams have a rough surface and good hydrophobicity due to the lotus effect (Figure 7c,d).

In addition, the microscopic structure of a MXene film can also be changed by external physical forces. Figure 7e shows the vertical alignment of MXene nanosheets achieved by the mechanical shearing of discotic lamellar liquid‐crystal phases of Ti_3_C_2_T*_x_*.[qv: 87] In this process, a nonionic surfactant, hexaethylene glycol monododecyl ether (C_12_E_6_), was introduced to increase the interaction between MXene nanosheets by the strong hydrogen bonding between the —OH groups of C_12_E_6_ and the —F or —O groups of the MXene nanosheets, resulting in an MXene lamellar liquid crystal (MXLLC). Subsequently, the 2D macroassembly was fabricated by applying a uniaxial in‐plane mechanical shear force to align the MXLLCs, followed by the removal of the extra components (Figure 7f–h). The prepared films have a shorter ion transport path and a larger number of available active sites than pristine MXene films because of their highly ordered parallel aligned structure, exhibiting an excellent thickness‐independent electrochemical performance up to 200 µm.

### 2.3 3D Macroassemblies

The 3D assembly of 2D materials from solutions is due to the phase separation induced by the formation of a 3D network sructure, while the solvent molecules serve as spacers or continuous templates to prevent the restacking of the 2D nanosheets.[qv: 85] As a result, the fabricated monoliths usually inherit the structural and key properties of the nanosheets and show many new excellent properties. Hence, it is of great importance to assemble 2D MXene nanosheets into 3D macroassemblies to broaden their applications.

As mentioned above, the 3D assembly of MXenes results in monoliths if the MXene nanosheets are assembled in an interconnected way with strong linkages, and this requires a uniformly distributed system to ensure effective assembly. Due to their superior hydrophilicity, MXenes can be uniformly dispersed in aqueous solutions, which is favorable for the construction of 3D networks with good functionality. That is to say, the development of 3D assembly from an aqueous solution plays an important role in the controllable assembly of MXenes for functional 3D monoliths.

Nevertheless, compared with graphene assembly, the 3D assembly of MXenes from solutions still faces many challenges, such as the product being relatively small in size, poor in sheet flexibility,[qv: 37] having a high stability of surface functional groups,[qv: 88] having a lack of crosslinking sites, and being prone to oxidation, etc.[qv: 89] To date, many efforts have been devoted to assemble 2D MXene nanosheets into 3D monoliths with a tailored structure by well‐designed processes.[qv: 35,37,45] According to the assembly mechanism, we divide these into two types, are crosslinking and template methods. We shall next discuss these two methods in detail.

#### 2.3.1 Crosslinking Method

Although it is difficult to achieve MXene 3D assembly because of their superior hydrophilicity caused by surface functional groups,[qv: 8] introducing a favorable driving force to counteract the hydrophilicity of MXene is the easiest of all the above challenges. Specifically, the hydrophilicity and hydrophobicity of MXenes can be balanced to achieve the 3D assembly of 2D nanosheets from aqueous solution, that is, balancing the interplane attraction and the electrostatic repulsion between the 2D layers to achieve phase separation. Among the driving forces, a crosslinker that connects adjacent MXene nanosheets plays an important role in the formation of 3D monoliths.

GO, with a large number of surface functional groups, assists the 3D assembly of low‐dimensional nanomaterials due to its good gelation ability.[qv: 90] As a result the 3D assembly of MXene nanosheets can be achieved with the assistance of GO using different interactions during the gelation process. The intimate interfacial interaction between the Ti_3_C_2_T*_x_* MXene and a rGO framework was demonstrated by Chen et al.[qv: 91] Driven by this interaction, Ti_3_C_2_T*_x_* can be incorporated into the rGO framework after a self‐convergence process, resulting in a Ti_3_C_2_T*_x_*‐based hydrogel. By the introduction of a reducing agent (NaHSO_3_) in the process, the GO was reduced to partially reduced GO and the oxidation of Ti_3_C_2_T*_x_* was prevented. Similarly, with the assistance of GO and ascorbic acid, the formation of a Ti_3_C_2_T*_x_*/rGO hybrid aerogel by a hydrothermal treatment followed by directional freeze‐casting has been reported, which is an efficient approach to construct highly conductive 3D Ti_3_C_2_T*_x_* porous structures.[qv: 92]

**Figure 8:**
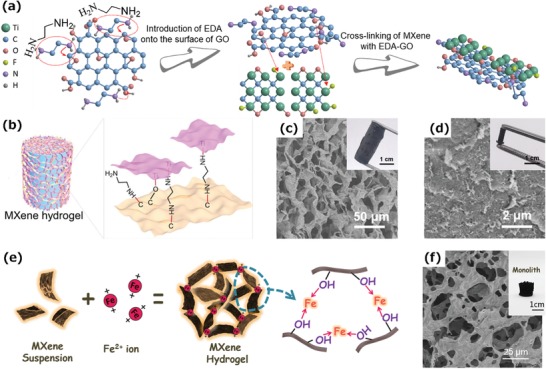
a) The formation mechanism of 3D MXene hydrogel. b) Schematic of the 3D MXene hydrogel. c) SEM image of MXene foams and inset an optical photograph. d) SEM image of dense MXene monoliths and inset an optical photograph.[qv: 37] e) Schematic of the metal ion‐initiated interaction of MXene nanosheets. f) SEM image of a MXene monolith and inset an optical photograph.[qv: 44]

The GO‐assisted assembly processes mentioned above mainly rely on intermolecular forces, such as van der Waals forces and hydrogen bonds to form a 3D hybrid MXene monolith, which frequently results in limited accessible surface area and more strategies need to be developed. Our group has reported a 3D MXene hydrogel (MXH) prepared by introducing chemical bonding between MXene nanosheets and rGO layers by a gelation process initiated by ethylenediamine (EDA) (**Figure** **8**a).[qv: 37] As a result, a robust hydrogel with high storage and loss modulus can be easily assembled from a Ti_3_C_2_T*_x_* aqueous dispersion even with a low concentration (2 mg mL^−1^) (Figure 8b). As a result of the stable interconnected framework, the MXH obtained can be further transformed into MXene foams (F‐MXM) or dense MXene monoliths (D‐MXM) with tunable pore structures by simply changing the drying method (Figure 8c,d), and these materials have important possible uses in the fields of pollutant removal and energy storage.

Another crosslinker, divalent metal ions, also initiates the fast gelation of MXene, resulting in a MXene monolith with a 3D network (Figure 8e).[qv: 44] Specifically, the divalent metal ions (such as Fe^2+^, Mg^2+^, Co^2+^, and Ni^2+^) rapidly break the electrostatic repulsion between the MXene nanosheets and serve as crosslinkers to bond them together. The bonding of metal ions and —OH groups reduces the hydrophilicity of MXene, leading to phase separation from an aqueous solution to initiate the gelation. As shown in Figure 8f, the prepared MXene monolith has an interconnected structure as well as strong enough linkages to form a monolith, which is attributed to the high binding energy between metal ions and —OH groups. It is worth noting that the gelation process is fast and works efficiently, thus preventing the MXene from oxidation during processing.

Other crosslinkers, such as EDA, cellulose, ammonia, and polyimide (PI) have also been proposed to assemble 2D MXene nanosheets into 3D macroassemblies.[qv: 35,47,93–95] Xing et al. fabricated a 3D MXene‐cellulose hydrogel, in which the cellulose served as a crosslinker to bond the MXene nanosheets together by covalent bonding as well as hydrogen bonding. The 3D MXene‐cellulose hydrogel had a favorable biocompatibility, outstanding photothermal properties, and a high loading capacity of the anticancer drug doxorubicin hydrochloride, while Wang et al. have demonstrated its excellent performance in supercapacitors.[qv: 47,95] In addition Liu et al. have reported the formation of an electrically conductive, superelastic, and lightweight MXene‐PI aerogel by the crosslinking effect of PI. It had a 3D macroscopic structure of MXene sheets.[qv: 35] In this study, poly(amic acid) was first used to assist the MXene assembly by hydrogen bonding, followed by heat treatment inducing the polymerization and forming a robust 3D macroscopic MXene‐PI structure. As a consequence, the MXene sheets were closely interconnected by forming a sandwich‐like structure and were constrained by the PI chains to form a robust 3D framework.

#### 2.3.2 Template Method

The template method, which can precisely control the shape and size of pores, is a widely used method for the preparation of functional materials, especially for the assembly of 2D materials into 3D monoliths.[qv: 96–100] Recently, the template method was also proposed for the 3D assembly of MXene nanosheets by several groups.[qv: 21,22,45,101–104] The templates generally used can be divided into two types, which are soft templates and hard templates, respectively.

**Figure 9:**
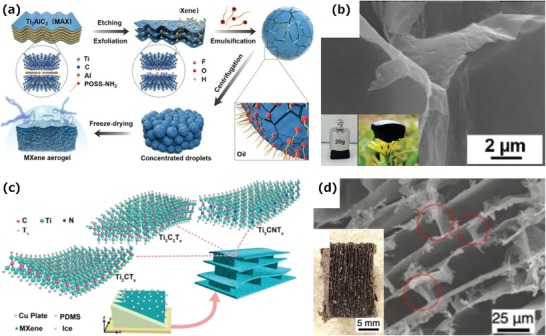
a) Schematic showing the construction of Pickering emulsions and MXene aerogels via MXene surfactants. b) SEM images of MXene aerogels prepared from emulsion templates. Inset: Optical photographs showing the robust, lightweight properties of MXene aerogels.[qv: 105] c) Schematic of the bidirectional freeze‐casting mechanism, and the aligned lamellar structure produced with interconnected bridges of MXene aerogels. d) SEM image of Ti_3_C_2_T*_x_* aerogels. Inset: Optical photograph of a free‐standing Ti_3_C_2_T*_x_* aerogel showing the orientation of the structure on the centimeter scale.[qv: 45]

Soft templates, such as micelles, emulsions, and liquid crystals have a low structural strength and low hardness. With a variable size and shape, soft templates are easy to fabricate and remove, thus becoming good candidates to assist the formation of 3D macroscopic structures with a tunable pore structure. Bian et al. fabricated a CTAB modified MXene with tunable hydrophilicity via electrostatic interaction, which wrapped the surface of emulsion templates to form a 3D framework due to the attached hydrophobic carbon tail.[qv: 101] Similarly, Shi et al. used oil soluble amine‐functionalized polyhedral oligomeric silsesquioxane (POSS‐NH_2_) to change the charge of oil–water interface from negative to positive, hence the negatively charged MXene nanosheets could assemble at the interface due to the electrostatic interaction. By homogenizing the Ti_3_C_2_T*_x_* dispersion and oil with dissolved POSS‐NH_2_, the water‐in‐oil Pickering emulsions act as templates to form an aerogel after freeze‐drying (**Figure** **9**a).[qv: 105] The as‐prepared MXene aerogels hold advantages of porous, hydrophobic, robust, and highly conductive (Figure 9b), which is favorable for the applications, such as oil adsorption and EMI shielding. The above two approaches pave a new way for preparing 3D MXene monoliths using a soft template.

In contrast, hard templates are usually solid materials with high structural strength and hardness. The 3D assembly of 2D nanosheets can be achieved by a combination of hard templates and 2D nanosheets using different interactions. Hard templates have a stronger regulatory effect on the assembly process and can precisely control the pore structure and size of the assembled materials. Typically, polymer spheres such as polystyrene (PS) and PMMA, are ideal hard templates for the 3D assembly of 2D nanosheets.[qv: 48] Sun et al. have demonstrated a highly conductive MXene@PS composite monolith, which was assembled using the electrostatic interactions between negatively charged MXene nanosheets and positively charged PS microspheres followed by compression molding to strengthen the overall structure.[qv: 102] The as‐prepared monolith had a high conductivity of 1081 S m*^−^* ^1^ as well as excellent EMI shielding performance.

Freeze‐casting is also an effective method to assemble 2D materials into a 3D structure by rapid freezing and ice crystal growth, which is known as the ice template method. By introducing ice templates, the structural strength of MXene/GO composites is increased by piling up the 2D nanosheets of GO and MXenes to form robust 3D networks, which gives the resulting monoliths excellent mechanical properties as well as high resilience.[qv: 22,103] The regulation of pore size and shape of 3D macroscopic structures can be achieved by controlling the conditions of ice crystal growth, such as the growth direction. Recently, a robust, compressible and lightweight aerogel with an aligned lamellar structure was fabricated by the bidirectional freeze‐casting of MXene colloidal solutions (Figure 9c,d).[qv: 45] In this process, temperature gradients in both the horizontal and vertical directions were achieved simultaneously, which led to the aligned lamellar structure of MXene flakes. With increasing concentration of the MXene colloidal solution, the interlamellar spacing decreased accompanied by the formation of “bridges” connecting adjacent MXene layers, which accounts for the excellent mechanical and EMI shielding performance. Much effort has been devoted to the assembly of a 3D network of MXene by freeze‐casting, such as MXene‐rGO or MXene‐based aerogels, which can be used in sensing, EMI shielding, and micro‐supercapacitors.[qv: 22,103,104]

## 3 The Applications of MXene Assemblies

As illustrated in Sections 2.–2.3.2. on MXene assemblies from 2D to 3D at the macro and/or microlevels, the assembled macroscopic materials not only have the intrinsic properties of MXene, such as a high electrical conductivity, varied surface chemistry, and high mechanical strength, but also have many novel advantages including lightweight, hydrophobicity, porous structures, and so on, which are favorable for many applications. Here we shall summarize the common applications of MXene assemblies including energy conversion and storage, EMI shielding and absorption. At the same time the relationship between the performance and the assembled structures will be discussed.

### 3.1 Energy Conversion and Storage

Supercapacitors and rechargeable batteries are the most promising electrochemical energy storage (EES) devices, whose performance mainly depends on the electrode materials.[qv: 106] Hence, developing new electrode materials is of vital importance and has attracted tremendous interest from many researchers. Following the discovery of Ti_3_C_2_T*_x_* and the breakthroughs in synthesis methods for MXenes, it was found that MXenes have great promise in constructing high‐performance electrode materials due to their excellent conductivity, large number of electrochemical active sites, etc.[qv: 15] Their electrochemical performance can be fully demonstrated using well‐designed assembly processes that overcome the restacking problems. MXene assemblies were first and mainly used in the field of supercapacitors, then extended to other fields of alkali metal ion energy storage. Here, the electrochemical performance of MXene assemblies in different energy storage devices is summarized in **Table** **1**.

**Table 1:** The Electrochemical Performance of MXene Assemblies in Different Energy Storage Devices

Material	Assembled structure	Device	C_s_ a)	Rate capability	Cycling stability	Ref.
C‐V_2_CT*_x_* (C = Li, Na or Mg)	2D assembly	SCsb)	420 F g^−1^ (5 mV s^−1^)	100 F g^−1^ (10 A g^−1^)	77% (100 A g^−1^, 1 000 000 cycles)	[qv: 33]
Hydrazine/Ti_3_C_2_T*_x_*	2D assembly	SCs	250 F g^−1^ (2 mV s^−1^)	210 F g^−1^ (100 mV s^−1^)	100% (5 A g^−1^, 1000 cycles)	[qv: 46]
MXene/SWCNT	2D assembly	SCs	390 F cm^−3^ (2 mV s^−1^)	350 F cm^−3^ (5 A g^−1^)	≈100% (5 A g^−1^, 10 000 cycles)	[qv: 43]
PPy/Ti_3_C_2_T*_x_*	2D assembly	SCs	417 F g^−1^ (5 mV s^−1^)	256 F g^−1^ (100 mV s^−1^)	92% (100 mV s^−1^, 25 000 cycles)	[qv: 42]
Ti_3_C_2_T*_x_*/PVA‐KOH	2D assembly	SCs	528 F cm^−3^ (2 mV s^−1^)	over 300 F cm^−3^ (100 mV s^−1^)	≈85% (5 A g^−1^, 10 000 cycles)	[qv: 56]
400‐KOH‐Ti_3_C_2_T*_x_*	2D assembly	SCs	517 F g^−1^ (1 A g^−1^)	210 F g^−1^ (100 mV s^−1^)	99% (1 A g^−1^, 10 000 cycles)	[qv: 57]
Ti_3_C_2_T*_x_*/PANI	2D assembly	SCs	371 F g^−1^ (2 mV s^−1^)	287 F g^−1^ (20 mV s^−1^)	98% (20 mV s^−1^, 10 000 cycles)	[qv: 58]
Ti_3_C_2_T*_x_* ionogel	2D assembly	SCs	70 F g^−1^ (20 mV s^−1^)	63 F g^−1^ (500 mV s^−1^)	80% (1 A g^−1^, 1000 cycles)	[qv: 75]
Ti_3_C_2_T*_x_*/rGO	2D assembly	SCs	1040 F cm^−3^ (2 mV s^−1^)	634 F cm^−3^ (1 V s^−1^)	100% (5 A g^−1^, 20 000 cycles)	[qv: 76]
Ti_3_C_2_T*_x_* hydrogel	2D assembly	SCs	1500 F cm^−3^ (2 mV s^−1^)	570 F cm^−3^ (2000 mV s^−1^)	90% (10 A g^−1^, 10 000 cycles)	[qv: 108]
Activated carbon/MXene film	2D assembly	SCs	126 F g^−1^ (0.1 A g^−1^)	71 F g^−1^ (100 A g^−1^)	92.4% (10 A g^−1^, 10 000 cycles)	[qv: 109]
V_2_C@Co	2D assembly	LICsc)	1117.3 mAh g^−1^ (0.1 A g^−1^)	199.9 mAh g^−1^ (20 A g^−1^)	100% (8 A g^−1^, 15 000 cycles)	[qv: 67]
CTAB‐Sn (IV)@Ti_3_C_2_	2D assembly	LICs	268 F g^−1^ (0.2 A g^−1^)	132 F g^−1^ (5 A g^−1^)	71.1% (2 A g^−1^, 4000 cycles)	[qv: 70]
PVP‐Sn (IV)@Ti_3_C_2_	2D assembly	LIBsd)	1375 mAh cm^−3^ (100 mA g^−1^)	504.5 mAh cm^−3^ (3000 mA g^−1^)	94.3% (500 mA g^−1^, 200 cycles)	[qv: 69]
Ti_3_C_2_T*_x_*/PEDOT	2D assembly	LIBs	307 mAh g^−1^ (100 mA g^−1^)	71 mAh g^−1^ (1000 mA g^−1^)	83% (100 mA g^−1^, 100 cycles)	[qv: 81]
Ti_3_C_2_T*_x_*/CNTs	2D assembly	SIBse)	421 mAh cm^−3^ (20 mA g^−1^)	89 mAh cm^−3^ (5000 mA g^−1^)	242 mAh cm^−3^ (50 mA g^−1^, 60 cycles)	[qv: 79]
MXene lamellar liquid crystal	2D macroassembly with micro 3D structures	SCs	270 F g^−1^ (10 mV s^−1^)	206 F g^−1^ (2000 mV s^−1^)	100% (20 A g^−1^, 20 000 cycles)	[qv: 87]
Ti_3_C_2_T*_x_*/rGO	2D macroassembly with micro 3D structures	LIBs	335.5 mAh g^−1^ (0.05 A g^−1^)	100.7 mAh g^−1^ (4 A g^−1^)	100% (1 A g^−1^, 1000 cycles)	[qv: 86]
Macroporous V_2_CT*_x_*	2D macroassembly with micro 3D structures	SIBs	470 mAh g^−1^ (2.5 C)	170 mAh cm^−3^ (25 C)	260 mAh g^−1^ (2.5 C, 1000 cycles)	[qv: 34]
MXene monolith	3D macroassembly	SCs	272 F g^−1^ (2 mV s^−1^)	226 F g^−1^ (1000 mV s^−1^)	97.1% (1 V s^−1^, 10 000 cycles)	[qv: 44]
3D MXene hydrogel	3D macroassembly	SCs	370 F g^−1^ (5 A g^−1^)	165 F g^−1^ (1000 A g^−1^)	98% (1000 mV s^−1^, 10 000 cycles)	[qv: 37]
Ti_3_C_2_T*_x_* aerogel	3D macroassembly	SCs	438 F g^−1^ (10 mV s^−1^)	349 F g^−1^ (2000 mV s^−1^)	90% (20 A g^−1^, 20 000 cycles)	[qv: 94]
Ti_3_C_2_T*_x_* /rGO aerogel	3D macroassembly	MSCsf)	34.6 mF cm^−2^ (1 mV s^−1^)	9.2 mF cm^−2^ (100 mV s^−1^)	91% (2 mA cm^−2^, 15 000 cycles)	[qv: 103]

a) Specific capacitance for SCs or capacity for LICs, LIBs, and SIBs

b) Supercapacitors

c) Lithium‐ion capacitors

d) Lithium‐ion batteries

e) Sodium‐ion batteries

f) Micro‐supercapacitors.

The performance of EES devices can be significantly improved by regulating the interlayer spacing to meet the needs of different energy storage systems,[qv: 107] which can be achieved by introducing external components between the layers. Typically, Ti_3_C_2_T*_x_* gel films in the presence of solvents between the MXene layers exhibited an ultrahigh volumetric capacitance of 1500 F cm^−3^ under a scan rate of 2 mV s^−1^, which is ascribed to the enlarged interlayer spacing thus giving access to more available active sites.[qv: 108] A cation‐driven assembled V_2_CT*_x_* film had a high gravimetric capacitance of 420 F g^−1^ at 5 mV s^−1^ with a smaller interlayer spacing but a highly stable structure due to the attraction of cations.[qv: 33] Other external components including polymers and nanomaterials are also mentioned above, and all show an improved electrochemical performance because of the elimination of restacking.[qv: 42,43]

**Figure 10:**
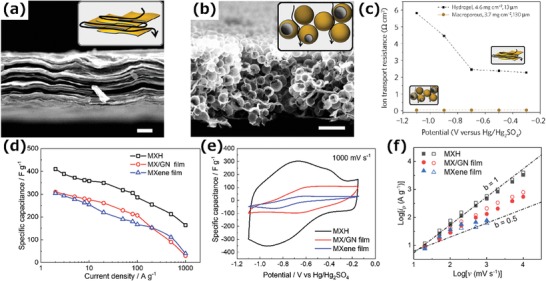
a,b) Cross‐section SEM images of a) a Ti_3_C_2_T*_x_* MXene hydrogel film and b) macroporous templated Ti_3_C_2_T*_x_*. Scale bars, 5 µm. Insets: The ionic current pathways in electrodes with different structures. c) Comparison of ion transport resistance for hydrogel and macroporous electrodes deduced from EIS measurements collected at different applied potentials. Reproduced with permission.[qv: 108] Copyright 2017, Springer Nature. d) Rate performance of the MXH, MX/GN, and MXene film electrodes at current densities ranging from 0.2 to 1000 A g^−1^. e) Cyclic voltammetry profiles of the MXH, MX/GN, and MXene film electrodes collected at 1000 mV s^−1^. f) Plots of the anodic (filled symbols) and cathodic (open symbols) peak current against the scan rate for MXH, MX/GN, and MXene films.[qv: 37]

Although introducing external components into MXene layers leads to a larger interlayer spacing for fast ion transport, the 2D layer structure still causes limited rate capability with increasing of electrode thickness because of the long ion transport paths (**Figure** **10**a). Hence, advanced MXene‐based structures, such as 3D interconnected networks, need to be developed for high‐performance electrodes. Gogotsi's group has demonstrated macroporous MXene electrodes produced using PMMA templates for EES devices that demonstrated an ultrahigh rate capability due to the significantly shortened ion transport paths (Figure 10b,c).[qv: 34,108] Similarly, the perpendicular alignment of MXene nanosheets mentioned above also results in much shorter ion transport paths than in a 2D structure, and shows thickness independent rate ability up to 200 µm.[qv: 87]

Recently, numerous studies have been devoted to assembling 2D MXene nanosheets into 3D macroscopic structures.[qv: 37,44,93,94,103] With a well‐tailored 3D network, the 3D MXene assemblies offer rapid ion transport paths and a high utilization of active sites, resulting in an excellent electrochemical performance. Because of this, novel 3D assembled MXene structures need to be more fully investigated to better understand and explore their energy storage applications.

The MXH electrode mentioned above showed a dramatic increase in capacitance and rate performance over MXene film, 2D MXene hybrid film, and reduced graphene oxide (MX/GN) film electrodes (Figure 10d,e).[qv: 37] The restacking of MXene nanosheets can be effectively restricted by forming a 3D interconnected network during the gelation process, which thus provides an interconnect network for fast ion transport and gives a high electrical conductivity. As a result, the 3D MXH electrodes exhibited an excellent gravimetric capacitance of 370 F g^−1^ at 5 A g^−1^ and an ultrahigh rate capability with a capacitance of 165 F g^−1^ even at 1000 A g^−1^ (Figure 10d). Moreover, as shown in Figure 10f, the b value of the MXH electrode is close to 1 for scan rates ranging from 20 up to 5000 mV s^−1^, demonstrating superior charge storage kinetics.

Additionally, our group has reported the excellent rate capability of a MXene monolith fabricated by metal ion‐initiated gelation, which is ascribed to the suppression of layer restacking and the construction of rapid ion transport channels. Nevertheless, there remain many challenges to the assembly of 3D macroscopic MXene structures for energy storage applications, including optimizing the pore structure of the macroassembly for a specific EES device, guaranteeing a large interlayer spacing of the MXene layers while assembling, preventing oxidation of the MXene sheets to ensure a high electrical conductivity, developing more assembly methods to increase the accessible surface area for electrolyte ions, etc.

### 3.2 Electromagnetic Interference Shielding and Absorption

Recently, MXenes have been widely used in EMI shielding and absorption due to their high electrical conductivity and polar surface caused by surface functional groups and defects.[qv: 110,111] Due to the well‐designed structures, many MXene assemblies show better EMI attenuation performance than pristine MXenes. In this section, we will discuss the uses of MXenes in EMI shielding and absorption from the assembly aspect.

A 2D layered film is the most common form of MXenes assembly. Typically, a 45 µm thick Ti_3_C_2_T*_x_*‐SA film had an EMI shielding effectiveness (SE) of 92 dB (>50 dB for a 2.5 µm thick), which is ascribed to the high conductivity and multiple internal reflections due to the expanded layered structure.[qv: 19] For the 2D MXene assemblies, the microwaves can easily penetrate the inner MXene layers, thus yielding multiple reflections causing conductive loss and polarization loss.

In addition to the 2D layer structure, core–shell structures are also widely used in electromagnetic wave attenuation. For instance, Ti_3_C_2_T*_x_* hollow sphere incorporated into a rGO skeleton can form freestanding monoliths, showing an excellent specific electromagnetic absorption performance value of over 14 299.2 dB cm^−2^ g^−1^ covering the whole X‐band at 3.2 mm.[qv: 20] At the same time, Ti_3_C_2_T*_x_*@PS showed a high conductivity of 1081 S m^−1^ and excellent EMI shielding performance of >54 dB over the whole X‐band with a maximum of 62 dB.[qv: 102] In the core–shell structure, high conductive components are generally coated on the surface of dielectric or magnetic particles for the construction of the conductive network, while the core provides high‐efficiency dissipation of electromagnetic waves, thus achieving excellent electromagnetic wave attenuation performance.

To achieve efficient electromagnetic wave attenuation, structures with a variety of interfaces and voids with different sizes have been proposed and widely used. For example, a rGO‐Ti_3_C_2_T*_x_* monolith constructed by directional freeze‐casting has an aligned cellular microstructure with a high electrical conductivity of 1085 S m^−1^, producing an epoxy‐impregnated composite with an EMI SE of more than 50 dB in the X‐band at a low Ti_3_C_2_T*_x_* content of 0.74 vol%.[qv: 92] Similarly, an anisotropic MXene aerogel with an aligned layer structure fabricated by bidirectional freeze‐casting showed an outstanding EMI SE of 70.5 dB.[qv: 45] In addition, 3D macroscopic structures produced by the crosslinking of MXene nanosheets also have porous structures, and are therefore promising for electromagnetic wave attenuation.[qv: 35,47] By improving the impedance matching between the void space and the free space, the structure significantly increases the number of reflections and scattering. At the same time, due to the dimensional resonance of electromagnetic waves, voids with different sizes lead to the broadening of the absorption bandwidth, resulting in an outstanding electromagnetic wave attenuation performance.[qv: 110]

Porous MXene foams have perfect impedance matching and high attenuation constants, and thus are good candidates for electromagnetic wave attenuation. As a typical example, a hydrazine‐induced MXene foam produced from a vacuum‐filtered MXene film had excellent properties of being hydrophobic, lightweight and flexible, and exhibited a high EMI SE of 70 dB.[qv: 18] Specifically, the pores formed by the foaming effect are matched with the free space impedance, which reduces the surface reflection of electromagnetic waves. The pore walls consisting of MXene nanosheets with good dielectric and magnetic properties can be used to dissipate the energy in the electromagnetic wave, thus achieving effective attenuation of electromagnetic waves.

### 3.3 Other Applications

In addition to the two above applications, MXene assemblies are widely used in other fields, such as sensing, environmental protection, catalysis, etc. Yue et al. reported a 3D hybrid porous MXene‐sponge network produced by a dipping‐coating method, followed by the insertion of insulating PVA nanowires as a spacer to construct a piezoresistive sensor, which exhibited a high sensitivity (147 kPa^−1^ for the <5.37 kPa region and 442 kPa^−1^ for the 5.37–18.56 kPa region), a low detection limit of 9 Pa, a rapid response time of 138 ms, and good stability over 10 000 cycles.[qv: 21] Similarly, Ma et al. fabricated an MXene/rGO aerogel that had a porous structure and combined the high specific surface area of rGO with the high conductivity of Ti_3_C_2_T*_x_*, thus exhibiting a high sensitivity (22.56 kPa^−1^), a fast response time (<200 ms) and good stability over 10 000 cycles.[qv: 22]

MXene assemblies can also be used in environmental protection. For instance, alkali‐treated MXene exhibited a preferential Pb^2+^ adsorption behavior due to ion‐exchange effects.[qv: 24] Our group has also demonstrated the high adsorption capacity of MXene monoliths for heavy metal ions (Pb^2+^, Cu^2+^, Zn^2+^, and Ni^2+^).[qv: 37] In addition MXene assemblies are good hosts for loading active substances for specific functions, such as photosensitizers, anticancer drugs and active materials in electrodes.[qv: 91,95,112]

## 4 Summary and Outlook

We have reviewed MXene assemblies from 2D to 3D at the macro and/or microlevels, including 2D assemblies, 2D macroassemblies with micro 3D structures and 3D macroassemblies. The corresponding assembly mechanisms and their applications have also been presented. Specifically, 2D assemblies, achieved by introducing external components between the MXene layers, have a layer structure and a tunable interlayer spacing. The 2D MXene assemblies possess many excellent properties, such as superior mechanical strength, high electrochemical activity, great chemical/physical stability, etc., all of which are favorable for building flexible devices, microenergy storage devices, etc. In addition, 3D assembly could introduce a 3D interconnected network and larger interlayer spacing to address the restacking problem of MXenes. The formation of 2D macroassemblies with micro 3D structures plays an important role in the construction of MXene functional materials because of their higher surface utilization with better functionality than 2D assemblies. Also, because of the difficulty of 3D MXene assembly, there are still few studies being devoted to constructing 3D macroassemblies, which has so for mainly included the crosslinking and template methods. The crosslinker can promote phase separation to form 3D MXene macroassemblies by introducing a favorable driving force to balance the hydrophilicity of MXenes, while the template method can precisely control the shape and pore sizes of MXene assemblies. The uses of MXene assemblies have been summarized in terms of energy storage, EMI shielding, and absorption together with other applications. The efforts on these applications‐oriented studies play an important role in promoting the longer‐term development of MXene assembly for materials with well‐designed structures and specific functions.

Although many of the studies on MXene assemblies reported so far have shown considerable progress to address the issue of the restacking problem, there are still many problems in designing desired MXene structures. For example, the matching of pore structure and corresponding applications, balancing the function exploiting after assembly and the intrinsic properties of MXene, further reducing the partial overlay of MXene sheets during assembly, improving our understanding of the MXene chemistry and its assembly behavior to reveal the basic principles governing the assembly and further realize precise control, exploring assembly principles for the monolayer assembly of MXene, extending the range of building blocks beyond Ti_3_C_2_T*_x_*, etc. The 3D assembly of MXene is more difficult than for other 2D materials like graphene and is still in an early stage of development. More effort needs to be devoted to developing 3D functional macroforms of MXene for the production of advanced devices. Given the promising properties of MXenes, we believe that there will be numerous novel MXene assemblies reported which will have great potential in many applications.

## Conflict of Interest

The authors declare no conflict of interest.
